# Image-based classification of plant genus and family for trained and untrained plant species

**DOI:** 10.1186/s12859-018-2474-x

**Published:** 2019-01-03

**Authors:** Marco Seeland, Michael Rzanny, David Boho, Jana Wäldchen, Patrick Mäder

**Affiliations:** 1 Institute for Computer and Systems Engineering, Technische Universität Ilmenau, Helmholtzplatz 5, Ilmenau, 98693 Germany; 2 Max-Planck-Institute for Biogeochemistry, Department Biogeochemical Integration, Hans-Knöll-Str. 10, Jena, 07745 Germany

**Keywords:** Plant identification, Deep learning, Zero-shot classification, Computer vision, Taxonomy

## Abstract

**Background:**

Modern plant taxonomy reflects phylogenetic relationships among taxa based on proposed morphological and genetic similarities. However, taxonomical relation is not necessarily reflected by close overall resemblance, but rather by commonality of very specific morphological characters or similarity on the molecular level. It is an open research question to which extent phylogenetic relations within higher taxonomic levels such as genera and families are reflected by shared visual characters of the constituting species. As a consequence, it is even more questionable whether the taxonomy of plants at these levels can be identified from images using machine learning techniques.

**Results:**

Whereas previous studies on automated plant identification from images focused on the species level, we investigated classification at higher taxonomic levels such as genera and families. We used images of 1000 plant species that are representative for the flora of Western Europe. We tested how accurate a visual representation of genera and families can be learned from images of their species in order to identify the taxonomy of species included in and excluded from learning. Using natural images with random content, roughly 500 images per species are required for accurate classification. The classification accuracy for 1000 species amounts to 82.2% and increases to 85.9% and 88.4% on genus and family level. Classifying species excluded from training, the accuracy significantly reduces to 38.3% and 38.7% on genus and family level. Excluded species of well represented genera and families can be classified with 67.8% and 52.8% accuracy.

**Conclusion:**

Our results show that shared visual characters are indeed present at higher taxonomic levels. Most dominantly they are preserved in flowers and leaves, and enable state-of-the-art classification algorithms to learn accurate visual representations of plant genera and families. Given a sufficient amount and composition of training data, we show that this allows for high classification accuracy increasing with the taxonomic level and even facilitating the taxonomic identification of species excluded from the training process.

**Electronic supplementary material:**

The online version of this article (10.1186/s12859-018-2474-x) contains supplementary material, which is available to authorized users.

## Background

Taxonomy is the science of describing, classifying and ordering organisms based on shared biological characteristics [1]. Species form the basic entities in this system and are aggregated to higher categories such as genera, families or orders depending on characteristics that reflect common ancestry. Each category in this system can be referred to as a taxon. Biological systematics uses taxonomy as a tool to reconstruct the evolutionary history of all taxa [2]. Historically, this aggregation was based on the commonality of specific morphological and anatomical characteristics [1, 2]. However, with the availability and inclusion of molecular data [3, 4] the view on phylogenetic relationships has been subject to a number of fundamental changes even on the level of families and orders, compared to the pre-molecular era [5, 6]. The evolutionary relationships underlying the phylogenetic tree which is reflected in current taxonomic system are not necessarily accompanied by apparent morphological relationships and visual resemblance. As a consequence, it is unclear whether images of plants depict visual characters that reflect the phylogenetic commonality of higher taxonomic levels.

A number of previous studies utilized machine learning techniques for automatic classification or recommendation of plant species [7–9] from images of flowers [10], leaves [11], or location and time of observations [12]. A recent study on image classification found that higher-level visual characteristics are preserved in angiosperm leaf venation and shape [13]. The authors used a machine learning algorithm based on codebooks of gradient histograms in combination with Support Vector Machines to classify leaf images into families and orders with an accuracy many times greater than random chance. The algorithm was found to successfully generalize across a few thousand highly variable genera and species to recognize major evolutionary groups of plants. Compared to holistic shape analysis, they demonstrated that leaf venation is highly relevant for higher-level classification. The study however had several limitations: it only targeted leaf venation and shape, the approach required expensive chemical pre-processing for revealing leaf venation, and all images required manual preparation for background removal, contrast normalization, and having a uniform orientation. Furthermore, with 5314 images constituting to 19 families and 14 orders, the investigated dataset was rather small. Motivated by the findings of this previous study, we aim to investigate whether taxonomic characteristics can also be discovered and learned from general plant images taken in natural habitats and varying in scale, perspective, and extent to which a plant is depicted. Using a broad set of plant species representing the angiosperm flora of Western Europe, we investigate achievable classification accuracy on the three taxonomic levels species, genera, and families, in order to answer the following research questions (RQ):

1. How is the classification accuracy affected by increasing intraclass visual variations as well as interclass visual resemblance when generalizing the taxonomic level from species over genera to families?
2. Can distinct visual characteristics of higher taxonomic levels be learned from species’ images in order to facilitate taxonomic classification of species excluded from training?
3. Which plant organs share visual characteristics allowing for correct taxonomic classification?

To answer these research questions, we investigated the classification performance of a convolutional neural network (CNN) trained on 1000 species belonging to 516 genera and 124 families. Contrary to the well curated images used in Wilf et al.’s study [13], we utilized plant images with a large variety in perspective, scale, and content, containing flowers, leaves, fruit, stem, bark, and entire plants. The images were not pre-processed, making our study representative for a real-life automated identification system. In a first set of experiments, we investigated whether the classifier becomes confused by an increasing visual variability when identifying taxa on the more abstract genus and family levels. In a second set of experiments, we investigated whether sufficient visual characteristics of a genus and a family can be learned so that even species excluded from training can be identified as members of the respective higher-level taxon.

## Results

### Identifying species, genera, and families (RQ 1)

**Fig. 1:**
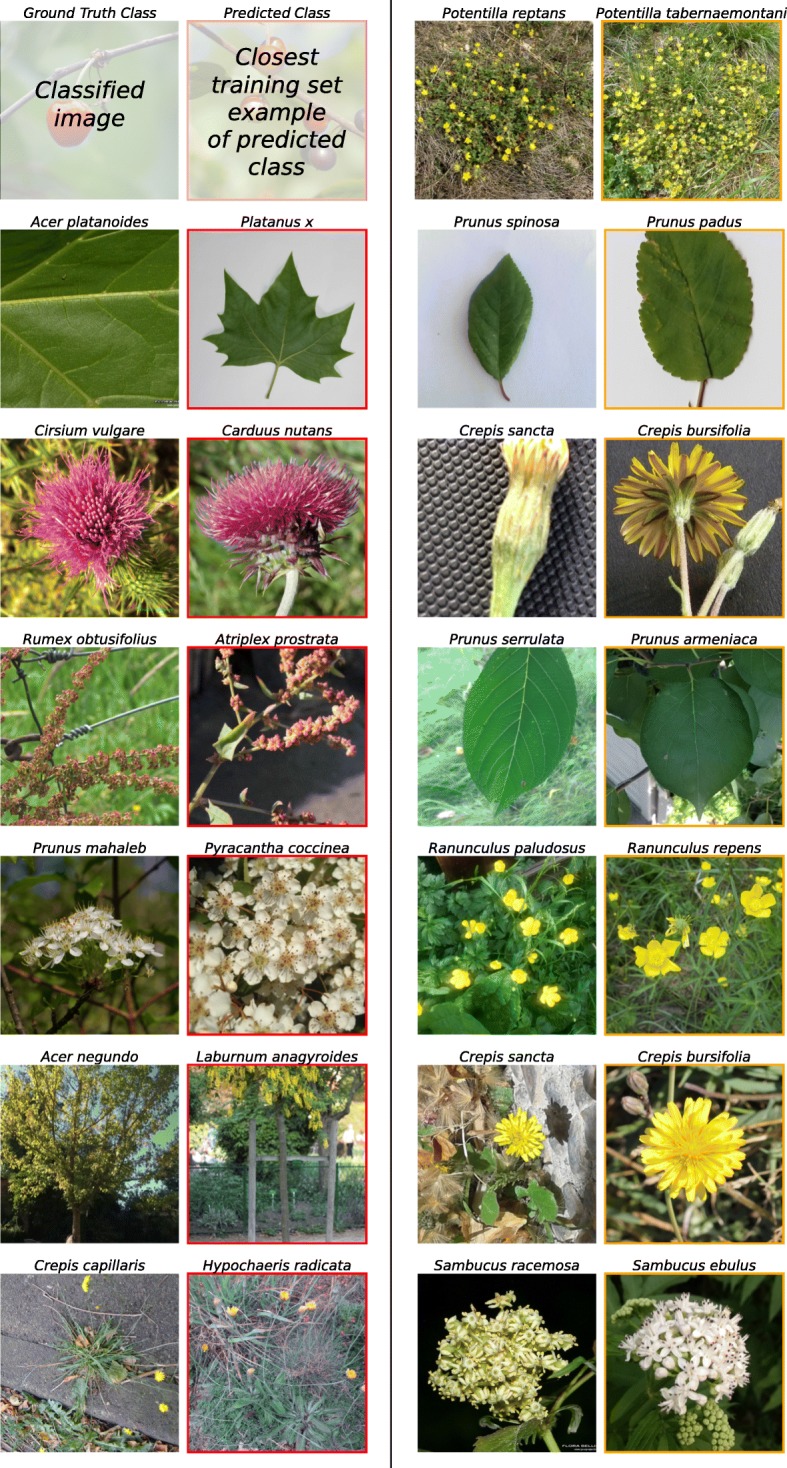
Examples of misclassified images. First and third column display the classified images, second and fourth column the predicted class. Red frames indicate wrong genus classification in hierarchy experiments, but correct direct classification at genus level. Orange frames indicate confusion with species of the same genus. Best viewed in electronic form

In an initial set of experiments we classified species, genera, and families on the full dataset. We applied the ’**inclusive sets**’ strategy (**InS**) with 90:10 partition. The “Methods” section provides details of the dataset, methods and strategies. We compared the results at genus and family level to hierarchy experiments. These experiments initially predict species. Then, corresponding genera and families are derived from the taxonomy and compared to the ground truth genus and family. Table 1 shows classification results on the three taxonomic levels in terms of top-1 accuracy, top-5 accuracy and standard deviation of the proportion of misclassified images according to binomial distribution. *N*_classes_ is the number of classes at each level and the suffix ’-H’ denotes the hierarchy experiments. Across the 1000 species in the dataset, the CNN classified 82.2% of the test images correctly (top-1). At the more general taxon levels, i.e., genus and family, accuracy improves relatively by 4.5% and 7.5%. For the hierarchy experiments, the accuracy improved relatively by 4.9% and 8.8% at genus and family level. For all experiments, the standard deviation showed a relative decrease of approximately 8% per level. The hierarchy experiments indicate that for 4% of the test images, species are confused with a different species of the same genus. For 7.2% of images, misclassified species are members of the same family. The remaining images, i.e., 13.8% at genus and 10.6% at family level, are classified as members of different genera and families, indicating high interclass visual resemblances and intraclass visual variations. The classifiers at genus and family level do not learn to separate them with higher precision, as indicated by the slightly larger accuracy of the hierarchy experiments. Examples of misclassifications are displayed in Fig. 1. Red frames indicate confusion with species from another genus, hence wrong genus classification in hierarchy experiments, but correct direct genus classification. Orange frames indicate confusion with species of the same genus.

**Table 1:** Classification accuracy at three different taxonomic levels using **InS**

Level	*N*_classes_	top-1 [%]	*σ*[%]	top-5 [%]	*σ*[%]
Species	1000	82.2	0.36	92.9	0.24
Genus	516	85.9	0.33	94.7	0.21
Family	124	88.4	0.3	96.5	0.17
Genus-H	516	86.2	0.32	94.7	0.21
Family-H	124	89.4	0.29	96.6	0.17

**Fig. 2:**
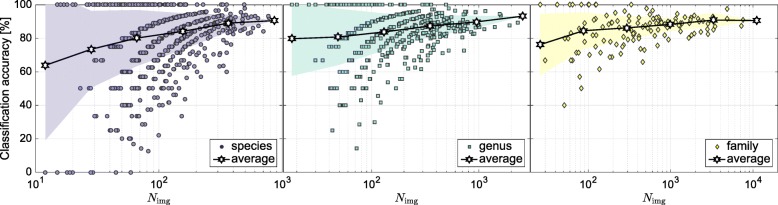
Class-averaged top-1 classification accuracy vs. number of images representing each species, genus, or family. Solid lines display the average accuracy and filled areas display the corresponding standard deviation

We further evaluated the dependency between classification accuracy and the number of images *N*_img_ representing each class. Figure 2 shows that the accuracy increased and the deviation in classification performance across taxa decreased with the number of training images. The deviation also decreased with the taxonomic level. The function-like characteristics of the accuracy for *N*_img_< 300 in Fig. 2 is affected by the dataset partitioning procedure, i.e., the test set is composed of 10% of the images per class (*N*_img, test_< 30), causing the class-averaged top-1 accuracy to be discretized depending on *N*_img, test_.

### Classifying genus and family of untrained species (RQ 2)

We performed another set of experiments at the genus and family level in order to study how well a CNN classifier can learn their visual resemblance and differences. We used the ’**exclusive sets**’ strategy (**ExS**), assuring that each genus and each family was represented by at least one distinct species in training and test sets. The total number of species *k*_S_ representing a class amounted to *k*_S_≥2. Table 2 summarizes top-1 and top-5 accuracy on both taxonomic levels. Each accuracy is an average across three experiments with random species selection following the **ExS** strategy. In comparison to the inclusive sets (**InS**), classification accuracy is reduced by more than half on the genus (55.4% relative) as well as on the family (56.7% relative) level (see Table 1).

**Table 2:** Three-fold cross-validated accuracy for classifying genus and family of untrained species in the exclusive sets **ExS**

Level	*N*_classes_	top-1 [%]	top-5 [%]
Genus	181	38.3 ±1.2	49.6 ±1.3
Family	81	38.7 ±3.4	48.0 ±3.0

**Fig. 3:**
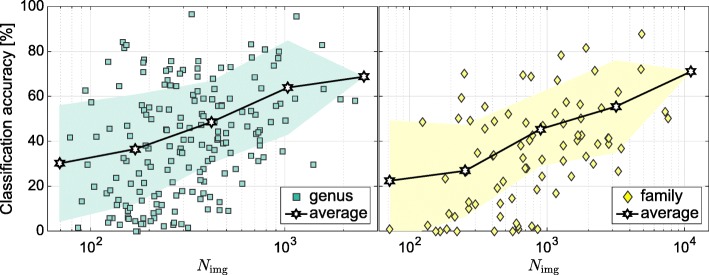
Class-averaged top-1 classification accuracy per number of images according to **ExS** strategy. The lines display the average classification, the filled areas the standard deviation

We evaluated the class-averaged classification accuracy with respect to the number of images representing each class (see Fig. 3). While the figure only provides an aggregated view across all genera and all families, the Supporting Information section contains additional tables on the accuracy per taxon. We observed that more images result in a classifier with higher accuracy, a trend similar to that observed for the **InS** experiments (cp. Fig. 2). However, we also observed a considerably higher variance in the trend. The achieved accuracy is not only influenced by the number of images, but also by the specific genus or family that was classified. Table 3 displays the five genera and families with best and worst classification accuracy.

**Table 3:** The five best and worst classified taxa at genus and family level according to the achieved top-1 accuracy on the ExS*

Level	Taxon	*k*_S,train_	*k*_S,test_	top-1 [%]	
				ExS	InS
Genus	*Orobanche*	5	1	96.5	93.3
(Best)	*Ophrys*	11	1	95.7	96.3
	*Arctium*	1	1	84.0	85.7
	*Viola*	8	1	83.2	84.1
	*Geranium*	12	1	82.9	94.7
Genus	*Linum*	1	1	2.2	85.7
(Worst)	*Diplotaxis*	1	1	1.6	87.1
	*Bartsia*	1	1	1.6	62.5
	*Gymnadenia*	1	1	0	76.5
	*Lychnis*	1	1	0	75.0
Family	Orchidaceae	41	5	87.6	97.4
(Best)	Geraniaceae	15	2	81.5	93.5
	Pinaceae	11	1	78.3	87.7
	Lamiaceae	43	5	72.0	91.5
	Betulaceae	8	1	71.5	89.9
Family	Lythraceae	1	1	0	89.1
(Worst)	Melanthiaceae	1	1	0	93.8
	Rutaceae	2	1	0	78.6
	Urticaceae	1	1	0	76.2
	Verbenaceae	2	1	0	80.0

*k*_S,train_ and *k*_S,test_ are the numbers of species in the dataset during training and test

^*^Results are three-fold cross-validated with random species selection during training and test

Successful classification using the **ExS** strategy is considerably more challenging since not the totality of their species, but the visual characteristics of families and genera need to be generalized and learned. Classification accuracy depends on the number of species representing a taxon during training (Table 3, 3rd column). For the **ExS** strategy, each classifier was trained on images of 90% of these species, e.g., five species for the genus of *Orobanche* and 41 species for the family of Orchidaceae but only one species for the genus of *Linum* and the family of Lythraceae. For 81 genera and 15 families the classifier was trained solely on images of one species and expected to classify another species of this genus or family (cp. Table 4), resulting in 28.7% and 12.6% accuracy respectively. These low accuracies are still 50 times (genera) and ten times (families) higher than random guessing with 0.6% for genera and 1.2% for families. In these cases, only if the overall visual appearance of two species is close and different to the appearance of other taxa in the training set, a high classification accuracy can be achieved. We found this applicable for the genus of *Arctium*, represented by *A. lappa* and *A. minus*, with an overall high visual resemblance. The genus of *Diplotaxis* on the other hand was represented by *D. tenuifolia* and *D. erucoides*. For >23% of the test images, the latter was misclassified as belonging to the genus of *Cardamine* due to the close resemblance of the inflorescence. The same applied to *D. tenuifolia*, which was regularly (20%) misclassified as belonging to the genus *Ranunculus*. The *Gymnadenia* species in the dataset, i.e., *G. conopsea* and *G. nigra*, were not recognized when training was conducted on only one of both species. A majority of authors consider the latter actually belonging to the genus *Nigritella*. The classifier also indicates their visual dissimilarity. It is a common phenomenon in plant systematics that different authors have different opinions on the membership of certain taxa [14]. We found that an increasing number of species and training images representing a genus or family yields an increasing classification accuracy. For instance, when only considering genera and families represented by at least three species (*k*_S_≥3), the average accuracy increases to 49.1% on the genus and to 39.1% on the family level.

**Table 4:** Number of species *N*_Spec_, genera *N*_Gen_, families *N*_Fam_ and total images *N*_img_ of the resulting dataset applying the **ExS** strategy at increasing minimum number of species *k*_S_ per genus or family

*k*_S_	*N*_Spec_	*N*_Gen_	*N*_img_	*N*_Spec_	*N*_Fam_	*N*_img_
1	1000	516	117713	1000	124	117713
**2**	665	181	**74013**	**957**	**81**	**110790**
3	503	100	55566	927	66	104786
4	407	68	45159	903	58	102315
5	295	40	32010	867	49	98756

*k*_S_=1 denotes the original dataset

*k*_*s*_=2 was selected for the **ExS** experiments

Among all families, Orchidaceae was classified best in the **ExS** experiments with 87.6% accuracy (97.4% for **InS**). Represented by 4873 images of 46 species belonging to 16 genera, this family is on the 4th rank of total images per class. The most frequent misclassifications for Orchidaceae were Plantaginaceae (2.6%) and Fabaceae (1.5%). This underlines the fact that the Orchidaceae species within the PlantCLEF2016 dataset represent a distinct and rather homogeneous group of species with similar appearance, different from species of other families. Hence, both the intraclass visual variability and the interclass visual resemblance are low for Orchidaceae. Orchids perform well because the images tend to resemble each other, with main focus on the flower, all resembling a similar habitusand a typical leaf form. The CNN learns these common traits from a broad set of species and is able to derive a generalized visual representation that allows to classify species excluded from the training process as members of Orchidaceae with an accuracy of 87.6%. Geraniaceae achieved the second highest accuracy (81.5%) in the **ExS** experiments, followed by Pinaceae (78.3%), Lamiaceae (72%), Betulaceae (71.5%), and Asteraceae (71.1%, not listed in Table 3). These families are well represented by a high number of species in the dataset (Asteraceae and Lamiaceae) or characterized by uniform and distinct physical appearance (Pinaceae, Lamiaceae). The species of these families also achieved high accuracy in the **InS** experiments.

Compared to the 81.7% classification accuracy achieved in the **InS** experiments, the classification accuracy of 38% for the Poaceae family was significantly reduced. The members of this family are characterized by a typical grasslike shape with lineal leaves and typical unsuspicious wind-pollinated flowers. The most frequent misclassifications involved Fabaceae and Plantaginaceae, of which some species from the latter family at least remotely resemble the appearance of Poaceae. We found it surprising that misclassifications as Cyperaceae or Juncaceae, two closely related families of the same order were virtually not present, although species of these three families are very similar in shape and appearance. This might be attributed to the content imbalance problem, i.e., differing distributions of image content categories during training and testing. We evaluated the negative impact of content imbalance on the classification accuracy in the Supporting Information (cp. Additional file 1: Figure S2). An explanation for the confusion with the dicotyledonous families might be that unlike most of the other families, the major proportion of the images refer to the content category “entire” where any specific traits are not distinguishable as the individuals are depicted from a distance in a clumpy or meadowlike manner. Eventually, grasses form the background of images of many other species. Very likely, this confused the CNN while learning a generalized representation of this family and caused the observed misclassifications. Potentially, structured observations along with defined imaging perspectives could increase the classification accuracy [8].

**Fig. 4:**
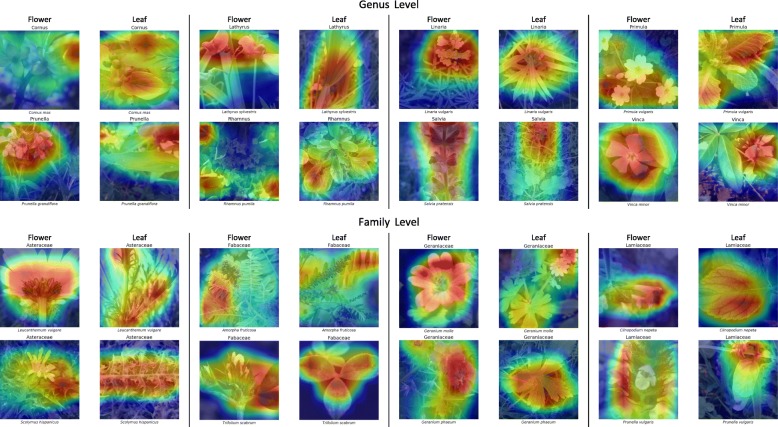
Image regions important for classification at genus (top rows) and family level (bottom rows). Best seen in electronic form

Given enough training images, the classifier successfully identified genus and family of trained species (**InS**) but more interestingly also of species excluded from training (**ExS**). To achieve these results, the classifiers learned distinct visual characters of genera and families. To visualize the reasoning of the classifiers on the test set images, we highlighted the neural attention, i.e., image regions responsible for classification, in terms of heat maps [15]. We manually evaluated several hundred images of genera and families. Representative images of flowers and leaves along with the neural attention at genus and family level are shown in Fig. 4. Most notably, the classifiers do not learn any background information. Instead, neural attention covers relevant plants or parts thereof. We observed that the classifiers often paid attention to characters such as leaf shape, texture, and margins, as well as attachment of the leaf. For some taxa, leaves seemed more relevant to the classifier if compared to flowers (cp. Cornus, Primula, Rhamnus, Fabaceae). For other taxa, flowers and inflorescence seemed more relevant than leaves (cp. Prunella, Salvia, Vinca, Geraniacea, Lamiaceae). Additional images covering more taxa are shown in the Additional file 1.

**Fig. 5:**
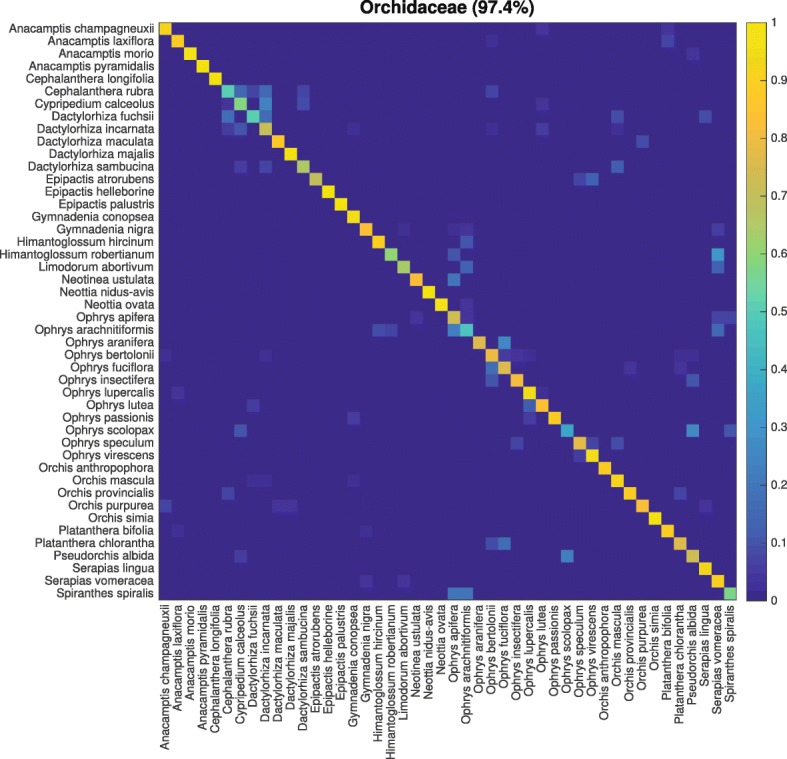
Confusion matrix for species prediction within the family of Orchidaceae

For genera and families with low intraclass variability and accordingly high classification accuracy on higher taxonomic levels, one may expect worse classification results on the species level. We selected the Orchidaceae family to study this phenomenon. Species in the Orchidaceae family are on average represented by 106 images and achieved 84.7% top-1 accuracy for the **InS** strategy, with a deviation of 14%. Figure 5 shows a confusion matrix on species level across all classifications within the Orchidaceae family. Only few species with high visual resemblance are prone to misclassifications and only 2.6% of the misclassifications belong to other families. In the same manner, we compared the classification accuracy of each family in the **ExS** experiments with that of its contained species in the **InS** experiments (see Figure S5 in the Additional file 1). We found a similar trend between accuracy at both taxonomic levels, i.e., few species from families with high resemblance can be confused. However, the effect is barely noticeable and the overall classification accuracy per family remains ≥60%. In result, we found that the CNN is able to do both, accurate fine-grained classification on species level as well as generalization to higher taxonomic levels.

### Plant organs sharing visual similarities (RQ 3)

Given the high visual variability at the genus and family level, we aim to understand the contribution of different plant organs in shaping the higher-level visual representation. Classification accuracy is increased if the plant organs exhibit distinct visual characters learned by classifier. Therefore, we evaluated the classification accuracy of the **ExS** experiments per image content category. The **InS** results imply that approximately ≥500 images per genus and ≥1000 images per family are required to learn the visual representation. This number of images is necessary as species with different appearance are merged into one class. The species themselves are represented by images with different content and at various scales and perspectives. As a result, the classes exhibit high visual variability at genus level and even higher at family level. The **ExS** results tell that higher-level taxa represented by many species achieved a higher classification accuracy when compared to taxa represented by only a few species (cp. Table 3). To take these aspects into account, we restricted the analysis of the **ExS** results to genera and families represented by at least five species and 500 (genera) respectively 1000 (families) images in the training set.

**Fig. 6:**
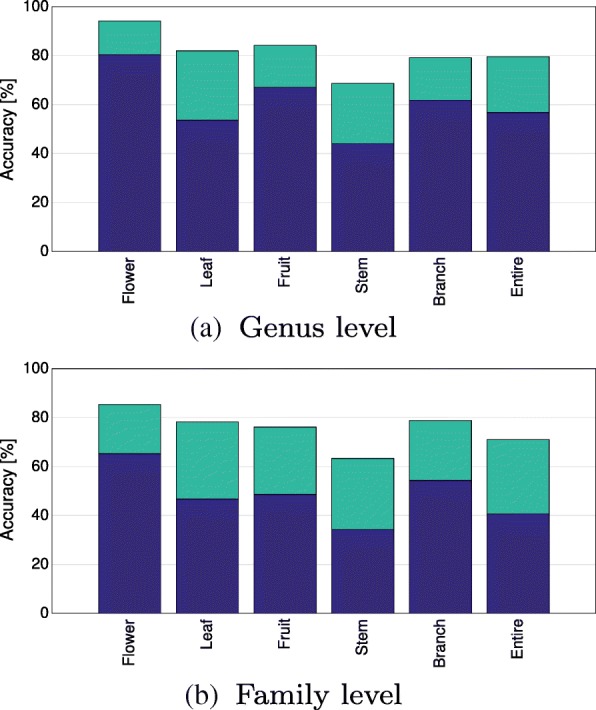
Averaged top-1 (blue) and top-5 (turquoise) accuracy for novel species grouped by image content for classifying **a** genera and **b** families

On average, flower images achieved the highest top-1 classification accuracy (cp. blue bars in Fig. 6) at both the genus (80%) and the family level (65%). Generally, all content classes on the genus level achieve better results than the content classes at the family level. The ranking of all content classes is identical for family and genus level, with images of the content class “entire” and “branch” forming the only exception. Leaf images achieved an overall lower accuracy than images depicting fruit. The content categories “entire” and “stem” achieved the lowest accuracy (cp. Fig. 6). Flower images also achieved the most accurate predictions on the genus level. Notable exceptions are the genera *Acer* and *Pinus*, where fruit and leaf images allowed for higher accuracy compared to flowers. Classification on fruit images achieved highest accuracy for the genus *Silene*. Also for classification at family level, flower images often yield the most accurate predictions. Notable exceptions are Asparagaceae and Boraginaceae, where images of stem and branch yield a higher degree of similarity. For Asteraceae, Fabaceae, Pinaceae, and Sapindaceae, fruit images performed best, i.e., 92.1%, 71.7%, 89.5%, and 62.4% in comparison to 83.5%, 64.5%, 72.7%, and 53.4% on flowers. For Fagaceae and Oleaceae, fruit and leaf images performed better than flower images. Detailed results per genus and family are given in the Supporting Information (cp. Additional file 1: Figure S6).

## Discussion

### Identifying species, genera, and families (RQ 1)

Wilf et al. stated that computer vision-based recognition is able to generalize leaf characters for predicting memberships at higher taxonomic levels [13]. Their study required a systematic, but time-consuming procedure for collecting images of chemically treated leaves. The authors achieved 72.14% classification accuracy on 19 families represented by ≥100 cleared leaf images. For this experiment, they used random sets without considering taxonomic membership, making their results comparable to our **InS** experiments. We used a CNN based classification pipeline and images with a large variability in quality and content. In this setting, we achieved 88.4% accuracy on 124 families, out of which 19 families were represented by ≤100 images. Our results demonstrate that despite sampling, content, and taxonomic imbalance (cp. “Image dataset” section), as well as high variability in viewpoint and scale, CNN-based image classification yields substantial performance for plant identification. Average top-1 species classification accuracy across the entire dataset was 82.2%, and increased with each taxonomic level relatively by 4% given the **InS** strategy (Table 1). The standard deviation showed a relative decrease of 8% per level. When confronted with highly variable and imbalanced data, the CNN benefits from an increasing amount of training data. For classes represented by ≤100 training images, the classification accuracy per class is moderate and yields on average ≈80*%*. For classes represented by ≥400 training images, class-averaged classification accuracy is consistently ≥70*%* per class and approaching 90% on average. Generalizing species to their genus and family level reduces the number of classes to be distinguished at the cost of increasing the intraclass visual variability and interclass visual resemblance. There are genera and families which form classes with large visual differences while species from different genera or families might resemble each other in specific organs. At species level, the lower intraclass variability caused 17.8% misclassifications. In 13.8% and 10.6% of these cases, the classifier confused species from another genus or family, as shown by the hierarchy experiments in Table 1. With misclassification rates of 14.1% and 11.6%, direct classification at the genus and the family level and was slightly less accurate. We attribute this to the increased intraclass variability and interclass resemblance along with the skewed data distribution intensified by taxonomic imbalance. With respect to our first research question, we conclude that:

1. When generalizing plant identification from species over genera to families, the classification accuracy shows a relative improvement of 4% per taxonomic level. Classification at these taxonomic levels is negatively affected by intraclass visual variability and interclass visual resemblance as well as taxonomic imbalance of the dataset. Taxonomic identification by species level classification is slightly more accurate.

### Classifying genus and family of untrained species (RQ 2)

We applied the **ExS** strategy specifically to evaluate classification accuracy on untrained species, i.e., species excluded from training at genus and family level. The strategy explicitly prevents a hidden species classification that could then be mapped to the family or genus and would bias results. A successful classification of genus or family implies that visual characters are shared among the species of the same taxonomic group. On exclusive training and test sets, Wilf et al. achieved 70.59% accuracy while classifying six families (≈4 times better than random chance). In our **ExS** experiments, an average classification accuracy of 38.7% was achieved for 81 families (≈32 times better than random chance).

We found the amount of training data necessary for learning visual representations to depend on the taxonomic level. While classification accuracy increased with a higher taxonomic level on the **InS**, the average accuracy decreased when classifying the genus and family applying the **ExS** strategy. Whereas 1000 training images per genus were sufficient to achieve a 60% average accuracy, the classification accuracy of families with 1000 images was less than 50% on average. Classification accuracy varied notably among different taxa in the **ExS**. The five best classified families reached accuracies of 71.5 to 87.6%, while the five best genera were classified with 82.9 to 96.5% accuracy. We conclude that distinct visual characters can be learned by a CNN in many cases even from a heterogeneous dataset. We also state that the classification accuracy is clearly linked to the number of species and images used for training and the intraclass visual variability. Hence, we conclude on our second research question:

RQ 2 Higher-level visual characters are preserved for many plant genera and families. Even from images with large variations in viewpoint, scale, and content, they can be learned by state-of-the-art classification methods. Sufficient amount and distribution of training images allow for taxonomic classification of species excluded from the training process.

### Plant organs sharing visual similarities (RQ 3)

Specific organs contain different amounts of visual information relevant for classification at higher taxonomic levels (cp. Figs. 4 and 6). For classifying excluded species in the **ExS** experiments, we found flower images to allow for the highest classification accuracy, i.e., 80% at genus level and 65% at family level. The accuracy achieved on leaf images were 25% (genus) and 20% (family) lower compared to flower images. This suggests a stronger preservation of higher-level visual characters for flowers than for leaves. Flowers consist of complex 3D structures with variation in shape, color, and texture. Their appearance from different perspectives hence contains complementary visual information which is beneficial for visual classification. Leaves on the other hand mainly represent 2D structures with rather homogeneous color space. For the vast majority of images they are depicted from their top side. Hence, the visual information is lower compared to flower images. For some images, it can be even difficult to isolate a single leaf as it is depicted as part of a mixture of different species and viewed from an arbitrary perspective and distance. Interestingly, the reduction of classification accuracy by classifying at family level instead of genus level was least for leaf images (54 to 46%). Despite leaf images often being prone to misclassification, this indicates that higher-level characters are also preserved in leaves. Stem images allowed for a classification accuracy of 43% and 34% at genus and family level. Visual inspection of stem images revealed that tree bark is classification relevant, e.g., for the family Pinaceae or the genus *Prunus*. However, for many stem images of herbaceous plants, leaves or flowers are additionally depicted in the image. This applies also to image categories “branch” and “entire”, where almost always leaves, flowers or fruit of the same plant are present on the image. Upon changing the classification level from genus to family, the accuracy is reduced by about 15%-25% for each image content category. We observe the strongest reduction for images of the category “fruit” and “entire”. This reflects the fact that overall shape and visual appearance of entire plants may differ strongly even among closely related species while flower and leaf shape are much more relevant for plant taxonomy.

Today’s taxonomy is based on genetic data expressed in a great variety of morphological characters. Some of these, e.g., the position of the ovary relative to the other floral parts, or the number of stamens per flower, are very specific and often constant for the members of a higher-level taxon. Many of such characters will hardly be discernible from the type of images present in the used dataset. The images are not standardized with respect to perspective, background and position. We may consider a number of causes for the differences in the achieved classification accuracy per taxon. Very likely, they are a consequence of resemblance regarding general shape, appearance and life form of the members in the sample. Obtaining high classification accuracy for families such as Orchidaceae and Pinaceae or similarly genera such as *Orobanche* and *Geranium* (Table 3) is linked to a low intraclass variability. This, on the other hand, is often connected to a similar perspective of the image. Other families such as Rosaceae comprise a much greater diversity of life forms and types of physical appearance, ranging from dwarf shrubs (*Dryas*) to bushes (*Rosa*) and trees (*Sorbus*). We conclude on our third research question:

RQ 3 Shared higher-level visual characters allowing for accurate classification at genus and family level are most dominantly preserved in plants’ flowers and leaves.

## Conclusion

We performed a series of systematic image classification experiments and studied the achieved accuracy across 1000 plant species belonging to 516 genera and 124 families. We used plant images taken in natural habitats with large variations in viewpoint, scale, and in the extent to which a plant is depicted. In a first set of experiments, we studied how a classifier can abstract from an increasing visual variability when identifying taxa on the more generalized genus and family levels. We found that CNN-based classification techniques are able to classify taxa on the genera and family level. However, the increase in classification accuracy per taxonomic level was found to originate mainly from a reduced number of classes to be distinguished. Grouping species at genus and family level forms classes with increased intraclass visual variability and interclass visual resemblance while intensifying data imbalance. Compared to species level classification, the classification accuracy was negatively impacted. The taxonomic identification of plants was found slightly more accurate if based on species level classification. In a second set of experiments, we investigated whether sufficient visual characteristics of genera and families can be learned so that even species excluded from training can be identified as members of such. We found that those species can be assigned to the correct high-level taxon for a broad set of genera and families. This implies that higher-level visual characteristics of genera and families are present for many taxa and that they can be learned by classification techniques, given sufficient amount and distribution of training data. Wilf et al. showed, based on images of cleared leaves, that plant systematic relationships are preserved in leaf architecture [13]. We argue that these relationships are similarly reflected in in-situ images, depicting a number of different plant organs. These images are of heterogenous quality and cover a much higher number of taxa. Future work on higher-level taxon classification from images should focus on improving data quality with respect to sampling and content imbalance, allowing to reveal and investigate the visual characteristics that facilitate a correct classification in more detail. Furthermore, taking taxonomic relations into consideration during classifier training and testing is a promising direction for advancing multi-label classification, which eventually allows accurate taxonomic identification at multiple levels using only one model.

## Methods

### Image dataset

**Fig. 7:**
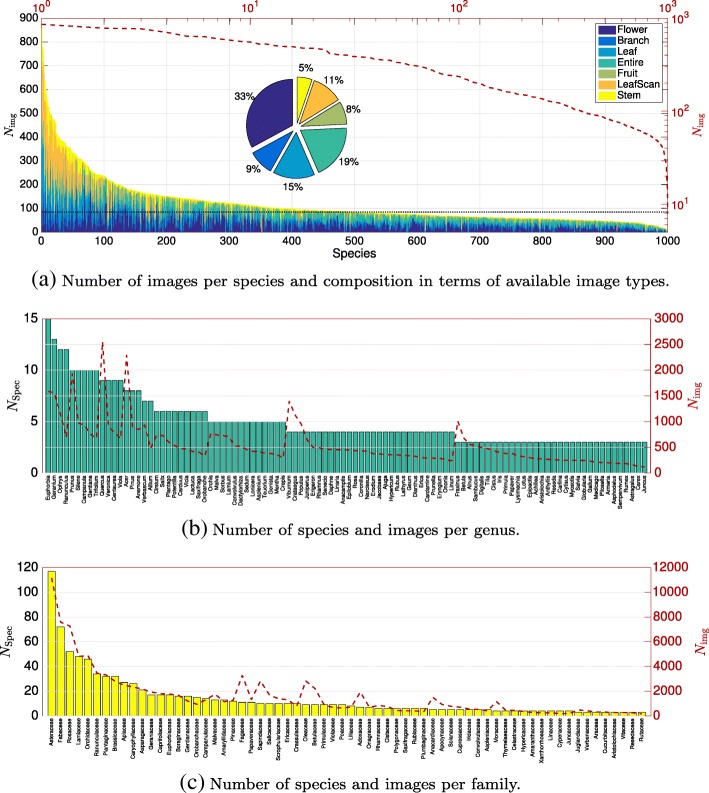
Distribution of images over taxa. **a** Number of images per species and content. The dashed red line displays the total number of images and the horizontal dotted line the median, amounting to 84 images. **b** and **c** display the number of species per genus and family. Please note that only 100 genera and 66 families represented at least by three species are displayed for sake of visibility

We utilized the PlantCLEF 2016 image dataset provided as part of the CLEF plant image retrieval task 2016 [16]. This datasets consists of 117,713 images belonging to 1000 species of trees, herbs, and ferns occurring in Western European regions. The images have been collected by 8960 distinct contributors of a citizen science initiative [7]. The plant families and genera occurring in this dataset reflect typical Western European flora. An accompanying XML file defines meta-data per image, i.e., an image category (namely flower, branch, leaf, entire, fruit, leaf scan, or stem) and the identified species name, including family and genus. Table 4 shows in row 1 the total number of species *N*_Spec_, genera *N*_Gen_, and families *N*_Fam_ present in the dataset. The dataset has three different sources of imbalance. In general, imbalance means that the frequency of occurrence of distinct classes within the dataset is highly skewed compared to a uniform distribution. Imbalance can cause low accuracy on underrepresented classes [17]. At species level, the number of images *N*_img_ per species varies from 859 (*Quercus ilex*) to eight (*Saxifraga media* or *Scilla luciliae*) with a median of 84 images per species (see Fig. 7a). As data was collected by a citizen science initiative, one source of imbalance is caused by the natural frequency and rareness of plant taxa in combination with geographical and seasonal sampling bias. Hence, we term this source **sampling imbalance**. The second source of imbalance relates to the image content categories. On average 33% of images display flowers, 26% leaves, and 19% the entire plant. The remaining images display branches (9%), fruit (8%), and stems (5%). This **content imbalance** causes biased classification if certain classes are primarily represented by images of a specific content category, e.g., flowers. Low classification accuracy can be expected if the test data is composed of underrepresented content categories. Targeting higher-level classification, the taxonomy adds a third source of imbalance, i.e., **taxonomic imbalance**. The number of species grouped into genera and families is highly different. Some taxa are hyperdiverse, e.g., the Asteraceae family which contains 117 species represented by 11,157 images, whereas others are monospecific, e.g., Lycopodiaceae with only one species and 26 images in total (cp. Fig. 7b and c). Even in case of balanced data at species level, taxonomic imbalance results in highly skewed distributions of images across higher-level taxa. The complete list of taxa, associated metrics, and the distribution of image categories across plant families are accessible via the Supporting Information.

### Model

We used convolutional neural network (CNN) based image classification. A CNN is a class of deep, feed-forward neural network with many layers, i.e., information processing steps. Each successive layer transforms the output of the previous layer. Starting from the raw pixel-based image as input to the first layer, the CNN learns filters that best translate input data into compact and discriminant representations for classification.

We chose the Inception-ResNet-v2 architecture [18] for our experiments, which combines the computational efficiency of inception modules [19] with the optimization benefits of residual connections [20]. Inception modules were engineered for efficient processing of information at different spatial extents [18, 19]. Residual connections, i.e., shortcuts in the model, allow for optimizing the residual of layer stacks and for training very deep neural networks with high accuracy [20]. The Inception-ResNet-v2 architecture achieved remarkable results on the ImageNet “Large Scale Visual Recognition Challenge” (ILSVRC) 2012 classification task^1^ validation set [21], i.e., 17.8% top-1 and 3.7% top-5 error [18]. The ILSVRC dataset consists of 1.4 million hand labeled images depicting 1000 object categories. We used TensorFlow, an open-source software library for large scale machine learning systems [22], for training and evaluation of the classifiers.

The number of parameters of CNNs is typically counting several millions, e.g., 54 million in case of the Inception-ResNet-v2. Hence, CNNs require a large amount of training data in order to avoid overfitting. A common strategy to avoid this is termed fine-tuning, i.e., to pre-train the CNN on a large dataset and then update the network weights by training on the actual data [23]. We used a network that was pre-trained on the ILSVRC data as available from the TensorFlow Model Garden [24].

For further augmenting the data during training, each image was pre-processed per epoch by selecting one random crop and applying horizontal flipping with random chance. Each random crop must cover at least 10% of the original image area while keeping an aspect ratio between 0.75 and 1.33. The resulting crop was then reshaped to fit 299 ×299 px and fed into the CNN for processing. It should be noted that by taking random crops, the classifier can potentially learn objects’ details to some extent. However, there is no guarantee that a crop displays the object of interest or part thereof at all.

### Experimental procedure

Seeking an answer for RQ1, our aim was evaluating classification accuracy on three taxonomic levels, namely at the species, the genera, and the families level. We trained a CNN on each level to classify the respective taxa, further denoted as ’classes’, and evaluated the classification accuracy. Per experiment, the image dataset needed to be partitioned into a training and a test set. We considered the authors’ original sets and found that the training set contained 113,204 images, while the test set merely contained 4509 images (<4*%*). Not only is the test set underrepresented, it only contains 495 of the 1000 species. Since we wanted to study results on the class level (species, genus, family), the original sets were insufficient for answering our research questions. We randomly created new class-based sets following a 90:10 partitioning, i.e., 90% of a class’ images are used as training set and the remaining 10% as test set. Since each class is represented in both training and test set, we refer to this partitioning strategy as ’**inclusive sets**’ or shortly **InS**.

In order to evaluate RQ 2 and RQ 3, we studied whether a CNN is able to learn a visual representation of a genus and a family in order to classify untrained species as belonging to that genus or family. These experiments required a different partitioning strategy: a species must only be present in either the training or the test set. Per genus and family, their species were partitioned 90% into the training and the remaining 10% into the test set. For genera and families with less than ten species, we ensured that at least one species was in the test set and the remaining were put into the training set. We refer to this strategy as ’**exclusive sets**’ or shortly **ExS**. The **ExS** requires a minimum number of species *k*_S_ representing a class (genus or family). On the one hand, increasing *k*_S_ allows a CNN to better learn a visual representation of a genus or family by abstraction across a larger variation within the species. On the other hand, the selection of *k*_S_ strongly affects the size of the remaining dataset, especially with respect to the taxonomic imbalance (cp. Fig. 7). Table 4 displays the remaining number of species, genera, families, and images, for an increasing number of minimal required species *k*_S_ per genus and family. For example, the last row of Table 4 shows that only 40 genera would remain in the dataset if we restrict our focus to genera with at least five species. We decided to set *k*_S_ to the smallest possible value, i.e., *k*_S_=2, for obtaining the largest possible dataset. That means that each class (genera and family) is represented by at least one species both in the training and the test set. Removing all genera and families represented by just one species resulted in two subsets of the original dataset: (1) for genus classification containing 181 genera represented by 665 species, and (2) for family classification containing 81 families represented by 957 species (cp. row 2 in Table 4). The difference between *k*_S_=2 and *k*_S_=3 in Table 4 shows that 81 genera and 15 families are represented by exactly two species. We applied threefold cross validation with random species selection for the **ExS** experiments.

### Training procedure and evaluation

In each epoch, a randomly cropped and flipped part of each image was trained in random order. The training loss was calculated as the cross entropy loss and a softmax function within the last layer. For mini-batches with a size of 32 images, the loss was aggregated and then propagated backwards in order to update network weights. The learning rate was set 5e-4 for the first 150,000 steps and 1e-4 for subsequent 100,000 steps. For testing, a single central crop (87.5% of the original image area) per image was forwarded through the network and classified. All experiments were evaluated in terms of top-1 and top-5 accuracy, averaged across all images of the test dataset. The top-*k* accuracy was computed as the fraction of test images where the ground-truth class label appears in the list of first *k* predicted class labels when predictions are sorted by decreasing classification score. The visual explanations, i.e., heat maps displaying neural attention, in Fig. 4 were computed using gradient-weighted class activation mapping [15].

## Additional file

Additional file 1 This archive contains spreadsheets with the complete list of taxa, i.e., species, genera, families, along with details on training and test set configurations and results for every taxon and experiment. Furthermore, the Supporting Information include evaluations on the impact of image content on classification accuracy and the **InS** at species level vs. **ExS** accuracy as well as additional neural attention visualizations. (ZIP 9011 kb)

## Abbreviations

CNN: Convolutional neural network
ExS: Exclusive sets
InS: Inclusive sets

## Footnotes

1 The data for the classification task remained unchanged since 2012.
