# Supplementary material for: Image-based classification of plant genus and family for trained and untrained plant species
Source: BMC Bioinformatics. 2019 Jan 3;20:4. doi: 10.1186/s12859-018-2474-x (PMC6318858; doi:10.1186/s12859-018-2474-x)
Supplement: Supplementary file 1 — This archive contains spreadsheets with the complete list of taxa, i.e., species, genera, families, along with details on training and test set configurations and results for every taxon and experiment. Furthermore, the Supporting Information include evaluations on the impact of image content on classification accuracy and the InS at species level vs. ExS accuracy as well as additional neural attention visualizations. (ZIP 9011 kb) [file 12859_2018_2474_MOESM1_ESM.zip › Supporting Information.pdf]

RESEARCH

Image-Based Classification of Plant Genus and Family for Learned and Unlearned Plant Species

Marco Seeland[1*], Michael Rzanny[2], David Boho[1], Jana Wäldchen[2] and Patrick Mäder[1]

*Correspondence:
marco.seeland@tu-ilmenau.de
[1]Institute for Computer and Systems Engineering, Technische Universität Ilmenau, Helmholtzplatz 5, 98693 Ilmenau, Germany
Full list of author information is available at the end of the article

Supporting information

Detailed set information and results

Detailed information on the data sets used for training and evaluation of every experiment are given in the following spreadsheets. The tables include the test/train configuration of the taxa used in every run, along with the resulting accuracy and evaluated confusion matrices.

InS-results.xlsx Details and results for the inclusive sets experiments.

ExS-results.xlsx Details and results for the exclusive sets experiments.

ExS-content results.xlsx Results for the ExS experiments evaluated per content.

Additional evaluation and figures

Difference in image set composition The content of every image can be one of seven distinct types: *flower*, *branch*, *leaf*, *entire*, *fruit*, *leafscan*, or *stem*.
Fig. 1 visualizes the content composition per family represented in the **ExS**.

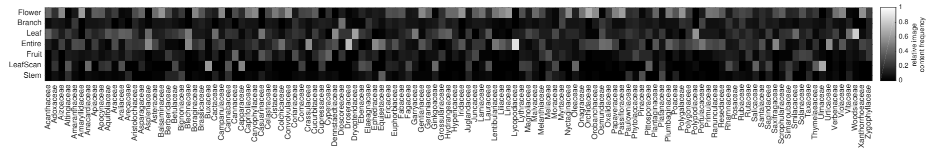

Figure 1 Relative frequency of images' content per family represented in **ExS** training and test sets.

Per genus and family, we evaluated the difference in composition of the **ExS** training and test sets, further denoted as content distance d, in terms of the amount of images per image content. Fig. 2 displays the classification accuracy versus the content distance d, computed as euclidean distance

$$d = \sqrt{\sum_{\text{content}} \left(\|v_{\text{training}}\| - \|v_{\text{test}}\|\right)^2}, \quad (1)$$

between the $L1$ normalized training and test set vectors v depicting the amount of images per content. The classification accuracy was found to depend on the image content and reduces with increasing content distance. Especially the accuracy for

families with comparably small amount of species (<3) and images (<1000) suffer from this dependency. Taxa with low visual similarity can be identified if both classification accuracy and content distance are low.

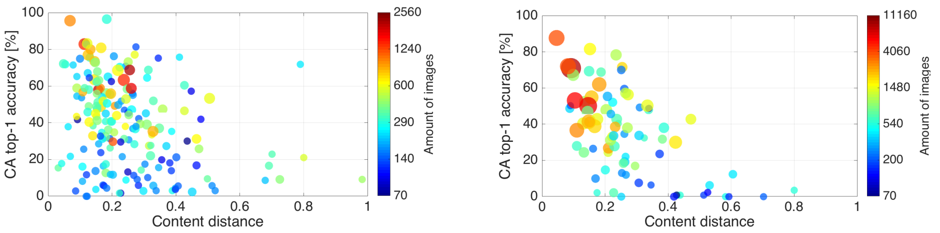

(a) Genus (b) Family

Figure 2 Average a) genus and b) family classification accuracy vs. the content distance of the training and test image sets. The dot size (color) relates to the amount of species (images) per genus/family.

Visual explanations at genus and family level In addition to Fig. 4 of the main article, Figs. 3-4 displays image regions responsible for classification using **ExS** for a broader set of genera and families.

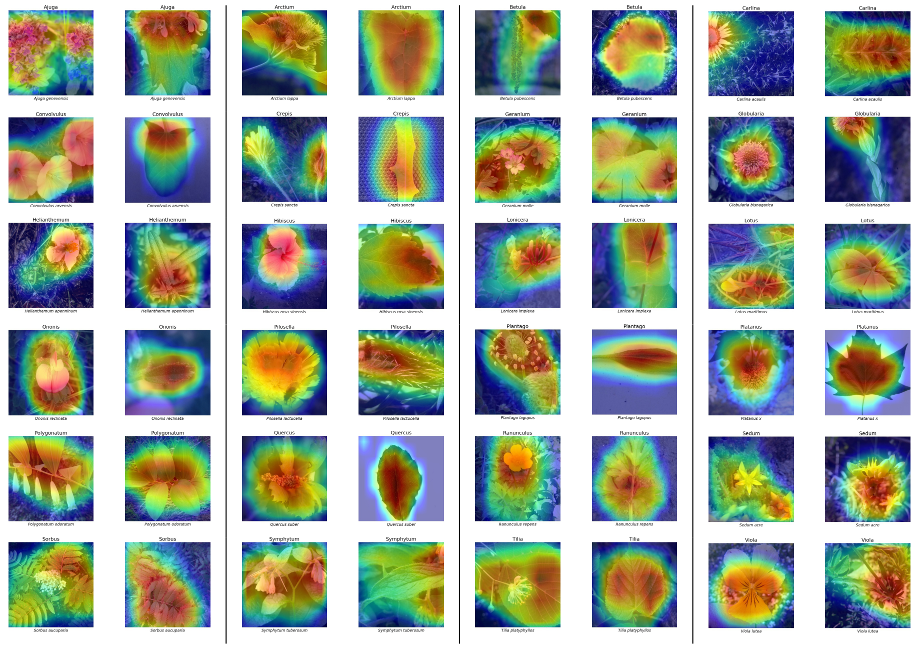

Figure 3 Image regions important for **ExS** classification at genus level. Best seen in electronic form.

Intra-taxon visual variation vs. inter-taxa visual similarity As detailed in the main article, we evaluated the average classification accuracy at species level resulting from the **InS** experiments for the species constituting to families investigated within the **ExS** experiments. Fig. 5 displays this average classification accuracy at species

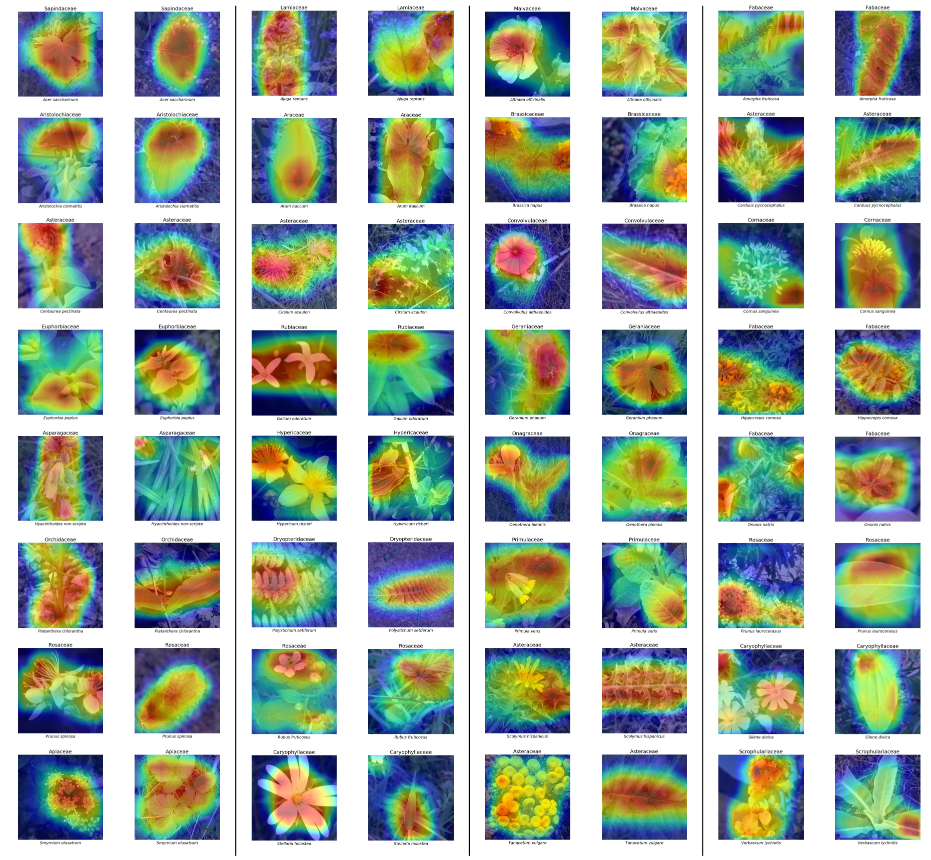

Figure 4 Image regions important for **ExS** classification at family level. Best seen in electronic form.

level, denoted as ⟨InS accuracy⟩, versus the classification accuracy of the corresponding family resulting from the **ExS** experiments.

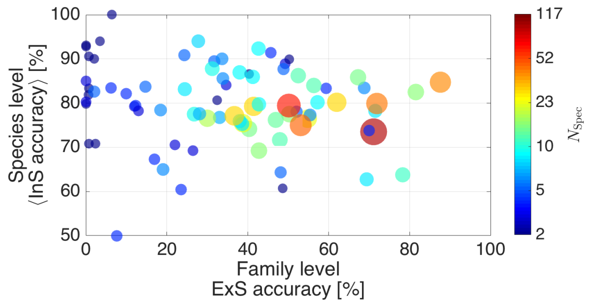

Figure 5 Classification accuracy per species applying the **InS** strategy versus the respective family classification accuracy applying the **ExS** strategy. The amount of species per family is depicted by the dot color whereas the dot size relates to the amount of genera per family. Please note the ordinate scale ranging from 50% to 100%.

***ExS** accuracy per class and content category* Fig. 6 displays the classification accuracy of the **ExS** experiments per class and content category. Three genera (*Euphorbia*, *Geranium*, *Prunus*) were recognized with an top-1 accuracy >80%. Eight

genera (*Campanula*, *Centaurea*, *Ophrys*, *Pinus*, *Quercus*, *Ranunculus*, *Salix*, *Verbascum*, and *Viola*) achieved top-1 accuracy of more than 90%. Five genera (*Acer*, *Anemone*, *Cirsium*, *Plantago*, and *Trifolium*) reached top-1 accuracies lower than 80%, but are amongst the top-5 in most cases. Two genera (*Gentiana* and *Silene*) achieved top-5 accuracies below 80%.

Author details
[1]Institute for Computer and Systems Engineering, Technische Universität Ilmenau, Helmholtzplatz 5, 98693 Ilmenau, Germany. [2]Max-Planck-Institute for Biogeochemistry, Department Biogeochemical Integration, Hans-Knöll-Str. 10, 07745 Jena, Germany.

References

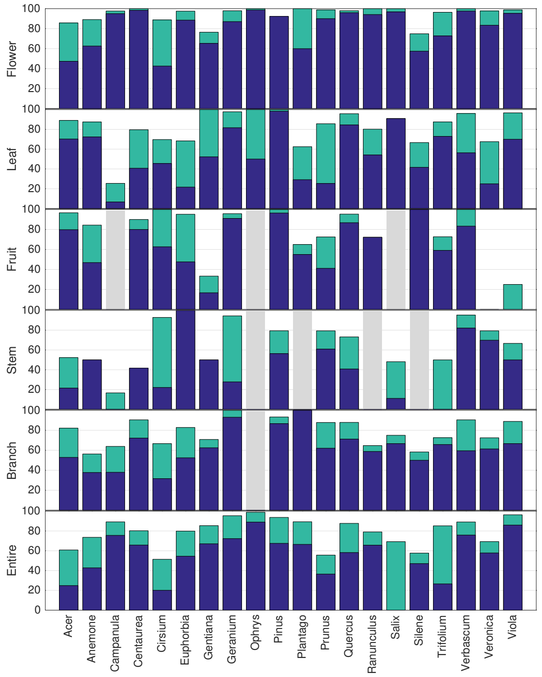

(a) Genus level

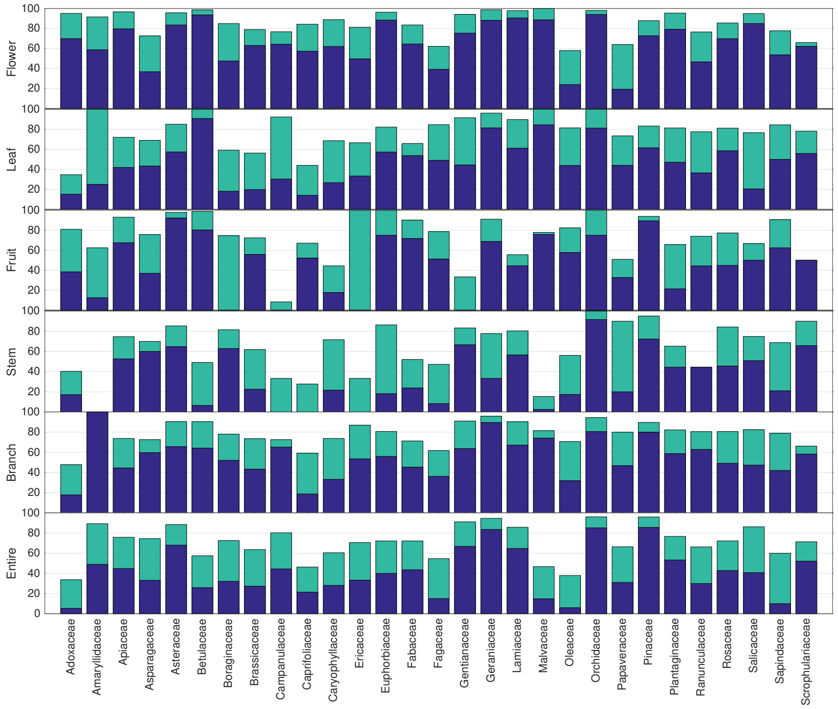

(b) Family level

Figure 6 Average top-1 (blue) and top-5 (turquois) classification accuracy per image content for a) genera and b) families represented by ≥500 images (≥1k for families) and ≥5 species during training. The grey bars indicate absence of test images for the combination of class and content.
